# Nanoparticle-Enabled Transdermal Drug Delivery Systems for Enhanced Dose Control and Tissue Targeting

**DOI:** 10.3390/molecules21121719

**Published:** 2016-12-15

**Authors:** Brian C. Palmer, Lisa A. DeLouise

**Affiliations:** 1 Department of Environmental Medicine, School of Medicine and Dentistry, University of Rochester, Rochester, NY 14642, USA; Brian_Palmer@URMC.Rochester.edu; 2 Department of Biomedical Engineering, School of Engineering and Applied Sciences, University of Rochester, Rochester, NY 14627, USA; 3 Department of Dermatology, School of Medicine and Dentistry, University of Rochester, Rochester, NY 14642, USA

**Keywords:** transdermal, drug, delivery, nanoparticle, nanocarrier, skin, disease

## Abstract

Transdermal drug delivery systems have been around for decades, and current technologies (e.g., patches, ointments, and creams) enhance the skin permeation of low molecular weight, lipophilic drugs that are efficacious at low doses. The objective of current transdermal drug delivery research is to discover ways to enhance skin penetration of larger, hydrophilic drugs and macromolecules for disease treatment and vaccination. Nanocarriers made of lipids, metals, or polymers have been successfully used to increase penetration of drugs or vaccines, control drug release, and target drugs to specific areas of skin in vivo. While more research is needed to identify the safety of nanocarriers, this technology has the potential to expand the use of transdermal routes of administration to a wide array of therapeutics. Here, we review the current state of nanoparticle skin delivery systems with special emphasis on targeting skin diseases.

## 1. Introduction

Transdermal drug delivery (TDD) offers many benefits, but it has proven difficult to discover drugs small and lipophilic enough to permeate effectively through the skin barrier. Transdermally delivered drugs often have better patient compliance than more painful/invasive alternative routes that require needle injection [1,2,3]. Transdermal routes also offer the benefit of bypassing the first-pass metabolism in the liver that orally delivered drugs undergo [1,2,3]. Drug formulations and concentrations can also be tuned to allow controlled release of drug into the body over long periods of time; thereby decreasing the need for multiple doses/applications [4]. For these reasons, TDD systems are an active area of research; however, few United States Food and Drug Administration (FDA)-approved transdermal drug formulations currently exist [4,5]. The first FDA-approved transdermally delivered drug was the scopolamine patch for motion sickness in 1979 [1,4,5,6]. Since then, a few other drugs like nicotine, fentanyl, estrogen, and testosterone have been successfully formulated into TDD systems [5]. Currently, the research goal is to use technology to enhance transdermal drug systems, and to discover novel methods to allow skin permeation of larger, hydrophilic drugs once thought to be impermeable to the skin.

There are many TDD systems being researched, including: microneedle injection, chemical penetration enhancers, physical barrier disruption by ultrasound or abrasion, and nanocarriers [1,7,8,9]. This review will focus on nanocarriers, which are particles made of polymer, lipids, or metals on the nanometer scale. These particles, if small enough, may penetrate into the viable layers of skin, and they can carry drug loaded on the particle surface or in the particle core. In many cases, nanocarriers allow deeper skin penetration and prolonged drug release compared to more traditional TDD systems [10]. While nanocarrier skin penetration can be limited in intact skin, these systems may be ideal for drug delivery through barrier-disrupted skin to treat diseases like psoriasis and atopic dermatitis (AD), two diseases characterized by chronic pruritic, inflammatory dermatitis and skin barrier disruption.

## 2. Skin Barrier

The skin is often called the largest organ in the human body, and it is a stratified structure comprised of two distinct layers named the epidermis and the dermis [11]. The skin has a number of important functions, including: physical barrier protection, immune surveillance, thermal regulation, ultraviolet light protection, and water retention. The epidermis, the outermost layer of skin, is responsible for both physical barrier protection from exogenous insults (pathogens, micron-sized particulates, and many large, hydrophilic chemicals) and water retention [12,13]. It is mainly comprised of keratinocytes, melanocytes, and Langerhans cells. The keratinocytes form the physical barrier of skin as they terminally differentiate from the stratum basale (lowermost epidermal layer) to the stratum corneum (outermost epidermal layer) [14]. The stratum corneum is comprised of physically dead keratinocytes called corneocytes; these dead cells are held together by a protein network (e.g., keratin, filaggrin, and loricrin) [14] and they are lined with a layer of fat (e.g., ceremides, fatty acids, cholesterol) [15]. The stratum corneum and the tight junctions in the stratum granulosum create a water tight barrier that is impermeable to most drug molecules that are both above 500 kDa and hydrophilic [16]. Melanocytes, also in the epidermis, provide melanin to the keratinocytes to absorb and block ultraviolet radiation from damaging the DNA in keratinocytes [17]. Langerhans cells survey the epidermis, and macrophages and dermal dendritic cells survey the dermis for pathogens and xenobiotic chemicals [18]. These cells can act locally, or they may travel to a skin-draining lymph node to activate an adaptive immune response via B or T cells [19,20]. Under normal conditions, the epidermis forms an adequate barrier for many environmental exposures, which makes designing drugs and nanocarriers that can penetrate into skin difficult. However, if drugs penetrate into the viable epidermis, they have access to living keratinocytes and immunologically active cells that could allow translocation of nanocarriers to draining lymph nodes. The epidermis is not vascularized and receives nutrients through diffusion, while the dermis contains numerous blood and lymphatic vessels. Therefore, nanocarriers that allow drug penetration beyond the epidermis and into the dermis may increase access to systemic circulation [21].

The dermis is comprised of three layers called the papillary dermis, reticular dermis, and hypodermis [22]. The upper dermis is comprised mostly of collagen and other extracellular matrix proteins, produced by fibroblasts [23]. The hypodermis is the lowermost layer that contains the subcutaneous fat [22]. The dermis is also comprised of a number of secondary structures like sweat glands, hair follicles, nerve fibers, and blood/lymphatic vessels [24]. Temperature is regulated by both hair follicles and sweat glands located in the dermis [25]. The secondary structures in skin and the papillary dermis create furrows and invaginations in skin that can trap topically applied drugs or nanocarriers [22,26]. These structures may act as a reservoir for drugs to slowly release into skin, but they may also allow increased drug penetration due to a decreased distance from stratum corneum to the dermis in these areas [27,28]. The blood vessels and lymphatics in the dermis allow many immune cells (macrophage, T cells, mast cells, and dendritic cells) to populate the dermis, but they also provide topically applied drugs with access to systemic circulation. As with scopolamine, the objective of most transdermal drug deliveries is to allow drugs to enter the dermis so they can diffuse into the systemic circulation [6]; however, in the case of skin diseases like psoriasis and atopic dermatitis, the goal is to get drugs into viable skin layers without entering systemic circulation to avoid off-target effects [29].

Researchers in the field of TDD are hoping to exploit skin structures like sweat glands, hair follicles, or skin furrows to enhance drug penetration and retention in the skin. There is also research on using penetration enhancers or physical abrasion to disrupt or damage the stratum corneum to allow increased penetration of nanocarriers. Lastly, nanocarrier design is heavily influenced by the stratum corneum structure with small nanoparticles (<100 nm) that either carry a charge to enhance flux through skin, or have a lipid coating to enhance retention in the lipid rich stratum corneum being highly favored [30,31,32,33]. As described in the following section, nanoparticle skin penetration is dependent on size, charge, morphology, and material composition. 

## 3. Nanocarrier Skin Penetration

In the last few decades, there has been an increase in the use of nanoparticles in consumer products. Nano-sized titanium dioxide and zinc oxide have been used since the 1990s in sunscreens and cosmetics to protect skin against harmful ultraviolet radiation [34], and more recently silica nanoparticles and fullerenes have been added to some cosmetic formulations to act as desiccants or free radical scavengers, respectively [35,36]. While the nanoparticles in these formulations are not intended to penetrate skin, their use signals a shift in the biomedical science and consumer product fields toward nano-enabled products. The increase in consumer products containing nanoparticles, and the research into nanoparticles as TDD systems has prompted many studies examining the effects of nanoparticles on skin toxicity. Many of these early studies examine skin penetration of nanoparticles in ex vivo or in vivo skin models, and they study the cytotoxicity of nanoparticles on in vitro skin cell models. The dogma for years has been that intact skin forms a barrier that is impenetrable by nanoparticles [37]; however, there is enough research in the field to suggest that nanoparticles can penetrate skin depending on size, charge, and material [38]. Beyond the dependence of skin penetration on size and charge, which is the focus of this section; nanoparticle dose, morphology [39], biological adhesiveness [40], and in vivo dissociation [41] can also play a role in skin penetration and biologic activity. 

**Figure 1:**
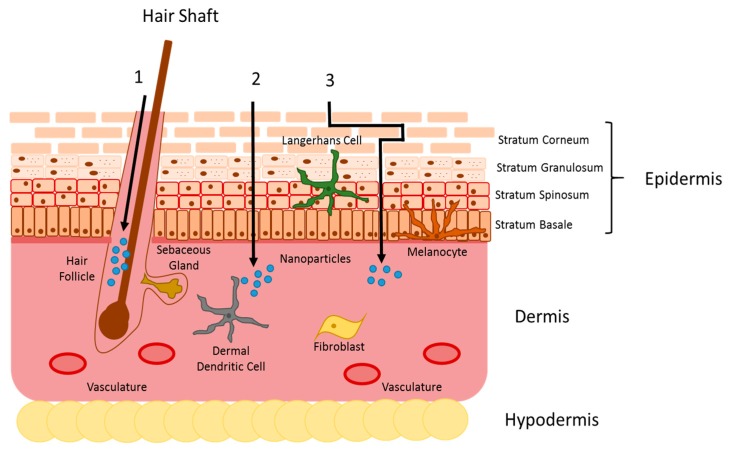
Illustration of nanoparticle skin penetration pathways. Topically applied nanoparticles can penetrate the skin in one of three different ways: (**1**) through the appendageal route, (**2**) through the intracellular route, or (**3**) through the intercellular route. The appendageal route involves nanoparticles entering hair follicles, sweat glands, or skin furrows for either penetration to the dermis or retention for increased drug release. The intracellular route involves a direct path through the cell membrane of multiple layers of the epidermis. The intercellular route involves a more tortuous path between epidermal cells. The pathway taken likely depends on the nanoparticle size, charge, morphology, and material.

Nanoparticles are observed to penetrate skin through one of three pathways: intracellularly through corneocytes, intercellularly around corneocytes, or via dermal structures like hair follicles (Figure 1) [42]. Work previously performed in our lab illustrates the penetration pattern for 30-nm cadmium selenide (core)/zinc sulfide (shell) quantum dots through hairless mouse skin after ultraviolet B radiation-induced skin barrier disruption [43]. The nanoparticles seemed to cluster in hair follicles and skin folds, and those quantum dots that appeared in the viable epidermis were observed in intercellular spaces. Our work and other research suggest that nanoparticles preferentially use the hair follicle and intercellular routes of penetration [43,44,45]. The Lademann group has shown a preferential trafficking of 320-nm dye-loaded particles into hair follicles of porcine skin [46]. Specifically, they identified that fluorescent dye loaded in microparticles preferentially accumulates in the hair follicles after massaging, compared to dye alone. The dye in the microparticles was detectable up to 10 days post application, illustrating the potential for long term drug reservoirs. Their work demonstrates that even large microparticles can aid TDD by facilitating drug nanocarrier accumulation, retention and drug penetration via the hair follicle. A major issue with the current nanoparticle skin penetration studies is the lack of consistent nanoparticles, animal models, application vehicles, and experimental design. In many cases, these discrepancies make comparisons between studies and extrapolation to human exposure conditions difficult. 

A standard tool for the study of skin penetration of small molecules and particles is the Franz diffusion chamber. In these studies, ex vivo skin is prepared either fresh or frozen, and the skin is placed into a device with both an upper and lower chamber. The particles are loaded into the upper chamber and after a period of time the lower chamber and the skin itself is tested for the presence of particles. F.L. Filon et al. demonstrated that 12-nm citrated gold nanoparticles penetrate damaged human ex vivo skin more so than intact human skin after 24 h of incubation in a Franz diffusion chamber [47]. However, C.S.J. Campbell et al. found no significant penetration of 20-nm polystyrene nanoparticles below the stratum corneum in intact or barrier-disrupted porcine skin in a similar Franz diffusion chamber set-up, after 16 h of incubation [48]. The differences in findings between these two studies are likely due to discrepancies in nanoparticle type and size, differences in skin preparation and source, or differences in the incubation times. Other ex vivo human or porcine skin studies have found that titanium dioxide nanoparticles measuring 50–100 nm remains either on the skin surface or within the stratum corneum in UVB-damaged porcine skin [49]; 70-nm silver nanoparticles penetrate into the viable dermis of porcine skin via hair follicles and translocate intracellularly [44], and dendritic nanoparticles measuring 140–160 nm penetrate into the viable epidermis in tape-stripped, barrier-disrupted ex vivo human skin [50]. While there is likely a reporting bias favoring publications displaying positive results for nanoparticle skin penetration, the results of many studies indicate that healthy skin is a formidable barrier to nanoparticle penetration beyond the stratum corneum but that penetration into the viable epidermis and systemic translocation can occur under certain circumstances. While human and even porcine skin are the best models for drug and nanoparticle skin penetration, it is often unethical or infeasible to use these models in large-scale toxicity and TDD studies. In these cases, rat and mouse skin, which is thinner than human skin and contains a higher hair follicle density [51,52], will suffice for an examination of in vivo nanoparticle penetration in worst-case scenario conditions.

The use of whole animal in vivo skin exposures allows more in depth pharmacologic and toxicological examination of the skin, and it also enables the study of whole animal tissue distribution of nanoparticles after topical administration. Our lab studied the effects of ultraviolet light B radiation-induced barrier disruption on skin penetration and tissue distribution of dihydrolipoic acid-coated 10–20-nm cadmium selenide (core)/zinc sulfide (shell) quantum dots with a negative surface charge. The presence of significant levels of quantum dots in the skin draining lymph nodes and the liver indicate the ability of the particles to penetrate through intact skin and enter systemic circulation, albeit in very low amounts compared to the total applied dose, <1% [53]. Tang Lei et al. confirmed this finding of quantum dot skin penetration and tissue distribution as well [54]. Other nanoparticles have been tested in similar rodent in vivo exposure models. For example, T. Hirai et al. examined the effect of very high dose (250 mg) amorphous silica nanoparticles applied to mouse ears over 3 days, and they found both skin penetration and nanoparticle translocation to skin-draining lymph nodes [55]. The elevated levels of nanoparticle penetration may reflect development of a dermatitis and consequent skin barrier defect from the application protocol over 3 days. In fact, in a mouse model of dermatitis induced by dithranol, 100-nm polymeric nanoparticles with either a positive or negative charge accumulated in damaged skin more than particles with a neutral charge [32]. Also, solid lipid nanoparticles 80 nm in diameter penetrated deep into rat skin, mostly via hair follicles [56]. Lastly, a study on the penetration of 20-nm titanium dioxide, commonly found in sunscreens, found minimal skin penetration through either intact or sodium lauryl sulfate-damaged skin [57]. 

Together, the literature on nanoparticle skin application demonstrates an ability of nanoparticles to penetrate skin in a number of circumstances. Also, in most cases skin barrier disruption-enhanced skin penetration or retention of the nanocarriers is tested. It is currently understood that nanoparticles <20 nm may penetrate or permeate intact skin, nanoparticles <45 nm may penetrate damaged skin [41], and larger particles may be translocated or stored in skin appendages [45,46]. Psoriasis and atopic dermatitis are two inflammatory skin conditions that are characterized by decreased skin barrier function [58,59]. These types of skin diseases are ideal candidates for use of nanocarriers to enhance drug skin penetration, retention, and targeting to allow better efficacy of current therapies, and also allow use of novel RNA-based therapies.

## 4. Psoriasis and Atopic Dermatitis

Psoriasis and atopic dermatitis are two pruritic, inflammatory skin diseases that lead to decreased skin barrier function, skin rashes or lesions, and psychosocial issues [58,59,60]. Atopic dermatitis leads to erythemic skin rashes, which are often infected with bacteria, like *Staphylococcus Aureus* [61]. Alternatively, psoriasis leads to erythemic skin rashes with plaques and scaling developing from abnormal stratum corneum arrangement and hyperproliferation of keratinocytes, and it is not usually associated with increased bacterial infections [62]. While psoriasis can develop at any age, atopic dermatitis normally presents during childhood, but it may persist into adulthood [63,64]. Psoriasis affects 2%–5% of the adult populations, most of which are the plaque or guttate psoriasis forms [65,66], while atopic dermatitis affects up to 20% of children in industrialized countries and an estimated 7% of the adult population [67]. There is no cure for either disease, and treatments are aimed at increasing barrier function, decreasing hyperproliferation of keratinocytes, and fighting bacterial infections [68,69,70,71]. While neither disease is life threatening, treatments can be expensive and the diseases carry a social stigma, since skin is a highly visible organ [72,73,74].

While the cause of atopic dermatitis is not completely understood, there are many predisposing factors in the pathogenesis of the disease, and currently the cause is thought to be linked to both genetic and environmental factors. Filaggrin (FLG), a key component of the cornified envelope of the stratum corneum that links keratin fibers, has a high rate (up to 47%) of mutation in many European atopic dermatitis patients leading to decreased skin barrier function [75,76,77,78]. Atopic dermatitis patients also have deficiencies in the tight junctions of the upper epidermis, specifically in the claudin-1 protein [79]. Deficiencies in skin barrier function may lead to increased environmental insults and bacterial infection, specifically *Staphylococcus aureus* infections, both of which are linked to atopic dermatitis [61,80,81]. Another hallmark of atopic dermatitis is an increase in T helper cell type 2 cytokines (interleukin (IL)-4, IL-5, IL-13), which leads to increases in immunoglobulin E (IgE) in serum [82,83,84]. Current therapies are aimed at decreasing the inflammatory response, repairing the skin barrier, and fighting bacterial infection [71]. 

Much like atopic dermatitis, the cause of psoriasis is largely unknown; however, it is linked to mutations in either the major histocompatibility complex (MHC) or the late cornified envelope (LCE) genes [85]. Similar to atopic dermatitis, psoriasis is also believed to progress or flare after environmental insult. The environmental insult may initiate the skin barrier damage, and psoriasis develops as keratinocytes hyperproliferate to repair the barrier. However, the keratinocytes fail to differentiate properly, leading to poor skin barrier formation and scaly plaque development [62,86]. Psoriasis also has an inflammatory component, but unlike atopic dermatitis, the inflammation is mediated by T helper Type 1 and Type 17 cells which increase cytokine levels in skin (IL-12, IL-17, IL-22) [82,87]. Likely due to differences in the inflammatory profile, psoriasis is not associated with bacterial infection [88]. The correlation of psoriasis with other autoimmune diseases has led researchers to question whether psoriasis is also an autoimmune disease; however, autoantigens in psoriasis have not been well characterized [89]. Currently there is a debate whether psoriasis is caused by direct action of autoantigens or whether the disease is initiated by a broader activation of innate immunity [90]. Existing treatment options target skin barrier repair and reduction of inflammation. 

Since both these conditions are inflammatory skin diseases, there is significant overlap in terms of treatment options. In both diseases, severe inflammation and barrier disruption may require systemic anti-inflammatory therapy (corticosteroids or cyclosporine), topical psoralen treatment with topical ultraviolet A (UVA) radiation (PUVA), or systemic treatment with biologically active compounds to block antibody or cytokine function [69,71,91]. However, this review will focus on topical treatments of skin diseases like topical steroids, retinoids, vitamin D derivatives, and calcineurin inhibitors. These drug classes are effective in decreasing dermatitis symptoms, but nanocarriers could improve treatment by increasing drug penetration and retention in skin, thereby reducing the number of applications needed, limiting drug concentrations, decreasing off-target effects, and potentially allowing the skin penetration of new classes of drugs and therapies. 

## 5. Transdermal Nanocarriers for the Treatment of Skin Diseases

There are many different types of nanocarriers being used in TDD research, and they can be broken down into three different categories: solid, liquid, or liquid crystalline phase nanocarriers [92]. Solid phase nanocarriers include metal core, solid lipid, and solid polymeric nanoparticles; and these particles tend to have the slowest drug dissociation. Liquid phase nanocarriers predominantly include micelles and nanoemulsions of lipids with a lower melting point than normal human body temperature, and due to their less structured lipid shell, they release drug much more readily than solid phase nanocarriers [92]. It has also been demonstrated recently that liposomes do not carry drug beyond the stratum corneum, but they potentially act as a penetration enhancer [93]. The less common liquid crystalline nanodispersions are normally synthesized using monolein, water, and poloxamer to create a fluid lipid nanoparticle that is more structured than traditional micelles [94]. Nanocarriers from all three classes are represented in the examples below. 

Currently, immunosuppressive calcineurin inhibitors are used in topical formulations to treat psoriasis and atopic dermatitis; however, skin penetration and off-target immunomodulatory effects are a concern. Tacrolimus is a calcineurin inhibitor that decreases T cell function in inflammatory skin diseases, and to increase penetration of the drug, Lapteva et al. demonstrated that loading tacrolimus into polymeric micelles of 10–50 nm allowed more drug to penetrate into the upper dermis of human ex vivo skin than drug without a nanocarrier [95]. The increased dermal penetration of tacrolimus was also demonstrated by Goebel et al. using a microemulsion system in human full-thickness skin [96]. Rodent models of skin disease also displayed a benefit of loading tacrolimus into a nanocarrier system. Applying tacrolimus therapy in a lipid nanoparticle measuring 20–100 nm, Pople et al. found increased skin targeting and dermatitis symptom reduction compared to drug without the nanocarrier, in a dinitrofluorobenzene (DNFB)-induced model of atopic dermatitis in BALB/c mice [97,98]. Lastly, Thapa et al. obtained similar results using liquid crystalline nanoparticles measuring 150–200 nm loaded with tacrolimus to treat imiquimod-induced psoriasis in a BALB/c mouse model [99]. Overall, there is greater dermal penetration, skin retention, and alleviation of dermatitis and inflammation when tacrolimus is delivered in a variety of nanocarriers. 

Corticosteroids are another commonly prescribed, topically applied treatment for both psoriasis and atopic dermatitis, due to their broad anti-inflammatory effects; however, long-term use can lead to off-target immunosuppression, skin atrophy, and irritation. Doktorovovo et al. displayed the capability to successfully encapsulate fluticasone propionate, a corticosteroid, into a nanolipid carrier system [100]. In the same year, Marchiori et al. loaded 200-nm polymeric nanoparticles with dexamethasone, in a hydrogel. They found that the in vitro drug release kinetics were more controlled when using the polymeric nanoparticles [101]. This early in vitro work using nanocarrier corticosteroid formulations led to subsequent in vivo efficacy studies. Fontana et al. used 200-nm lipid core nanocapsules to deliver clobetasol propionate, and they found that when treating Wistar rats with nickel sulfate-induced dermatitis, there was a slower drug release compared to drugs without the nanocarrier [102]. More recently, Eroglu et al. used liposomes measuring 220–350 nm to apply betamethasone valerate and diflucortolone valerate to DNFB-induced atopic dermatitis skin in Wistar rats [103]. They observed similar therapeutic effects when compared to currently available corticosteroid creams that contain 10 times the dose applied in the nanocarriers. Lastly, Siddique et al. used positively charged polymeric chitosan nanoparticles (230 nm) to deliver hydrocortisone and hydroxytyrosol to the skin of healthy Wistar rats. They observed increased skin targeting and decreased systemic uptake the drugs when compared to traditional, non-nanocarrier formulations [104]. These examples illustrate the more beneficial drug release kinetics and decreased drug concentration requirement when using nanocarriers. 

Retinoids are another class of topically applied drugs commonly prescribed alongside a corticosteroid to treat psoriasis. While retinoids are more commonly used to treat acne vulgaris, retinoids like tazarotene are prescribed to decrease inflammation and abnormal hyperproliferation of keratinocytes in psoriasis patients. Ourique et al. first displayed the possibility to encapsulate the retinoid tretinoin into a polymeric nanocarrier system [105]. They later observed an increased skin accumulation of tretinoin and decreased transdermal delivery when the drug was encapsulated in the polymeric nanocarrier [106]. Tretinoin has also been successfully formulated into solid lipid nanocarriers with limited in vitro keratinocyte cytotoxicity [107]. Other groups have illustrated an increased skin accumulation of tretinoin when applied in a liposome of 100–150 nm containing a penetration enhancer [108]. Raza et al. studied a number of tretinoin nanocarrier systems (<200 nm) and examined both the skin penetration and anti-psoriatic activity of the drug formulations. They found that all carrier systems (liposomes, solid lipid nanoparticles, ethosomes, and nanostructured lipid carriers) increased skin penetration of tretinoin, compared to commercial creams. However, only nanostructured lipid carriers and liposomes containing tretinoin increased orthokeratosis in a mouse tail model of psoriasis, compared to the commercial cream [109]. While research into nanocarriers for corticosteroids, tacrolimus, and retinoid is relatively new, nanocarriers could potentially decrease drug costs by reducing either the drugs concentration or application interval, or minimize toxicity by increasing skin retention and targeting of drugs. However, beyond enhancing currently available therapeutics, nanocarriers offer the potential for new drugs to be used in the treatment of skin diseases.

Some drugs are reserved for severe cases of atopic dermatitis or psoriasis, since they need to be taken orally, and they have many adverse side effects like immunosuppression and organ toxicity. Methotrexate is an oral drug that is used in severe cases of psoriasis to decrease lymphocyte function. This drug is large and hydrophilic; however, Bessar et al. used 4-nm gold nanoparticles coated with methotrexate to enhance skin penetration and keratinocyte uptake of the drug [110]. The in vitro skin penetration of methotrexate was also observed to be greater when the drug was loaded in lipid-based nanocarriers [111,112]. Singka et al. examined the permeation of 100-nm nanogels with sodium carbonate-mediated release of methotrexate, and an increase in methotrexate permeation across porcine skin was observed [113]. More recently, Avasatthi et al. used 278-nm nanostructured lipid carriers to deliver methotrexate in a mouse model. They observed slower, more prolonged release of the drug, and they revealed that the drug nanocarrier system decreases the psoriatic area and severity index, compared to drug alone in an imiquimod-induced mouse model of psoriasis [114]. Cyclosporin A is another oral drug used to inhibit T cells and induce immunosuppression in severe cases of both atopic dermatitis and psoriasis. Romero et al. demonstrated that 350-nm cyclosporin A polymeric nanoparticles could penetrate through barrier defected porcine skin, potentially allowing better TDD than bulk cyclosporin A [115]. Kim et al. also loaded cyclosporin A into 73-nm solid lipid nanocarriers, and they observed increased in vitro mouse skin penetration. The cyclosporin A-loaded nanocarrier also decreased IL-4 and IL-5 in an ovalbumin-induced mouse model of atopic dermatitis [116]. These examples demonstrate the ability of nanocarriers to enhance penetration of the larger, hydrophilic drugs, and on-going research into these novel therapies will ensure their future use to treat severe cases of inflammatory skin disease and to better target the drugs to skin compared to the oral delivery route.

The literature also contains examples of therapies not currently used to treat inflammatory skin diseases, but used in nanocarriers to treat animal models of skin disease. For example, ketoprofen, a non-steroidal anti-inflammatory drug, was loaded into 200-nm chitosan nanoparticles and used to treat an imiquimod-induced model of psoriasis in C57BL/6 mice [117]. The researchers observed an increased penetration of drug into the skin, decreased skin thickness, decreased transepidermal water loss, and decreased IL-17 and IL-23 release when compared to drug without the nanocarrier [117]. Another potentially novel treatment for skin diseases are ceramides, a component of the lipid structure in healthy stratum corneum. Jung et al. successfully loaded ceramides into 200-nm chitosan nanoparticles and used them to treat a sodium dodecyl sulfate (SDS)-induced model of atopic dermatitis in rats [118]. Following treatment with ceramides in the nanocarrier, they observed stratum corneum repair in the rat model. Alternatively, silver ions are well known for their anti-microbial effects, and Keck et al. used 200-nm nanolipid complexes with electrostatically bound silver ions to display anti-microbial and anti-inflammatory effects in a DNFB-induced mouse model of atopic dermatitis [119]. Lastly, some nanoparticles may have immunosuppressive effects when applied without drugs [120]. Shershakova et al. applied nC60 fullerenes topically to an ovalbumin-induced mouse model of atopic dermatitis, and they observed decreased IgE release, decreased cytokines, and better histological outcomes [121]. A similar effect has been shown using nanosized zinc oxide nanoparticles; however, while the nanoparticles could decrease swelling associated with ovalbumin/staphylococcal enterotoxin B-induced atopic dermatitis in the mouse, the zinc oxide led to increased levels of IgE [122]. Work currently under review in our lab that also illustrates an ability of small negatively charged nanoparticles (20–150-nm silica nanospheres, quantum dots, or gold nanoparticles) to reduce skin inflammation and swelling in a murine model of contact dermatitis. Beyond currently approved therapies, there are many drugs and particles that could effectively manage skin inflammatory diseases. 

Lastly, targeted biological inhibition of genes involved in skin disease progression is a fairly new, but very promising line of research. Currently only a few proof of concept experiments have been performed to display small interfering ribonucleic acid (siRNA) skin penetration and targeted gene silencing. The group of Zheng et al. was one of the first to show siRNA skin penetration into mouse and human skin when applied to a 13-nm gold nanocarrier [123]. They also observed the targeted silencing of the epidermal growth factor receptor (EGFR) gene in the epidermis and upper dermis. Since then, other groups have looked at more targeted approaches in psoriasis treatment [124]. These researchers have again demonstrated the skin penetration of siRNA loaded nanocarriers and the targeted knockdown of the human beta-defensin 2 encoding gene, a protein known to be highly expressed in the skin of psoriasis patients [125,126]. Using a reconstructed human skin in vitro model of skin inflammation, another group has demonstrated that IL-6 targeted siRNA loaded into liquid crystalline nanodispersions (200–270 nm) can penetrate into the upper dermis and decrease the extracellular release of IL-6 [127]. Most recently, Kanazawa et al. have used a protein based nanocarrier to deliver RelA (p65 subunit of Nuclear factor kappa-light-chain-enhancer of activated B cells (NF-κB)) siRNA to a mouse, and they observed decreased ear swelling in a DNFB-induced NC/Nga mouse model of atopic dermatitis [128]. Since both psoriasis and atopic dermatitis have been linked to gene mutations, a targeted, individually designed therapy may be used to treat these diseases by either targeting inflammatory or skin barrier-linked genes.

Nanocarriers represent a promising area of TDD for the treatment of skin disease due to the observed increase in drug skin penetration and skin targeting, the potential for decreased drug concentration or application intervals, and the addition of new therapies previously unused due to low transdermal penetration (Table 1). However, more research is needed in this field of nanocarriers and skin disease. Many studies are proof of concept type studies which simply display the ability of nanocarriers to enhance penetration of drug into the skin or retention of drug in the skin. Many of the studies examined used in vivo models of skin disease; however, in most instances the models more appropriately modeled irritant or allergic contact dermatitis rather than psoriasis or atopic dermatitis. Psoriasis and atopic dermatitis have both genetic and environmental triggers, which create more complicated disease phenotypes than those modeled by application of a chemical sensitizer. More stringent testing of nanocarriers, using genetic animal knockout models of psoriasis and atopic dermatitis, is warranted in the future, to better extrapolate the data to potential human use.

## 6. Discussion

Nanocarriers are being researched for many applications in TDD, since delivery through skin is less painful than injection, bypasses first pass metabolism in the liver, and can be formulated for slow and constant release of drug into systemic circulation. For these reasons nanocarriers have been investigated for use in transdermal delivery of vaccinations [129], anti-hypertensive drugs [130], antiparkinsonian drugs [131], and chemotherapeutics [132]. However, healthy skin forms a physical barrier to xenobiotic chemicals and particulates, and as illustrated here, nanoparticles may penetrate intact skin in a manner dependent on size, charge, and material. Nanoparticle penetration is also enhanced by skin barrier disruption; therefore, nanocarriers are highly suited to treating skin diseases like psoriasis and atopic dermatitis, which harbor skin barrier defects. 

Psoriasis and atopic dermatitis can be physically and psychologically debilitating diseases that together affect over 10% of the adult population [65,67], and the economic burden of psoriasis alone is over $50 billion annually in the US [73]. Current therapies are only effective in reducing symptoms, and nanocarriers could have the benefit of enhancing the efficacy of currently used drugs and decreasing potential off-target effects. Beyond using nanocarriers to enhance current therapies, newer types of topical therapies including large, hydrophilic drugs could be added to the skin disease therapy repertoire. For example, methotrexate, cyclosporin A, ceremides, and ketoprofen were all able to penetrate skin when applied using a nanocarrier. Many of these drugs can have serious off-target effects including systemic immunosuppression and organ toxicity, which means their use is limited to severe cases. Nanocarriers allow skin targeting of the drugs, which decreases off-target effects and potentially decreases toxicity.

While there is the potential for decreased drug toxicity due to increased skin targeting and retention, there needs to be extensive, chronic toxicity testing of these nanocarriers and drugs before any human testing. As of 2014 there were 43 nano-enabled pharmaceuticals on the market [133], and while that does not include any treatments for psoriasis or AD, nanocarriers are expected to play a large role in the treatment of skin inflammatory diseases in the future. The most readily marketable nanocarrier-based drugs may be tacrolimus or corticosteroids, since they are already approved for topical delivery. However, there is limited relevant nanoparticle skin toxicity research in the literature, since the studies that do exist are mostly in vitro studies that examine high doses of nanomaterials. As an example, a review of titanium dioxide nanoparticle toxicity focused more on pulmonary and gastrointestinal toxicity than dermal toxicity. The only studies examining titanium dioxide dermal toxicity tested for either acute skin irritant effects or in vitro cytotoxicity [134]. In the nanocarrier literature reviewed here, only two-thirds of the articles mentioned nanocarrier toxicity. Most articles referred to previous work using in vitro models, while some performed their own toxicity screening in vitro using either human fibroblasts or keratinocytes, and examining cell death as the only metric [103,107,110,118,123,124,127]. Some researchers also examined gross toxicity and potential irritant effects [95,96,112], and Pople et al. performed the most comprehensive toxicity assessment with a 28-day toxicity study that examined multiple target organs for histological abnormalities [98]. While all these studies found no skin irritant effects and no significant cytotoxicity, more in vivo toxicity assessments should be performed, since in vitro dosimetry is different compared to in vivo topical exposure. In vitro exposure also removes potential cellular interactions and signaling that exist in vivo, like potential immunomodulation as a result of the nanocarriers [135]. Our own lab has demonstrated the capability of nanoparticles to either suppress or stimulate inflammation in a contact dermatitis model, dependent on the nanoparticle size, type, and surface charge. While this effect could potentially be exploited in certain therapies [121], nanocarriers should be studied for potential immune stimulation or suppression [120]. The drugs approved for oral delivery, like methotrexate and cyclosporin A, need to be evaluated for dermal toxicity as well, since the concentrations in skin may be vastly different when comparing oral versus topical drug delivery. Nanocarriers could benefit the treatment of skin disease, but nanocarrier toxicity testing and drug efficacy testing in relevant animal models must be completed before any clinical use. While the dermal toxicity of nanocarriers is not the focus of this review, future nanocarrier research should include more stringent toxicity analyses, especially when the nanocarrier formulation has not previously undergone toxicity testing. 

The growing use of nanocarriers to transdermally deliver siRNA into skin for knockdown of targeted genes related to skin disease is promising. Not only does this demonstrate the ability to allow skin penetration of larger macromolecules into skin with the aid of nanocarriers, but it also demonstrates a shift in the field toward targeted genetic therapies. Therapies like these could be tailored to individual patients to maximize therapeutic outcomes. While this will not provide a cure, it could have an added benefit in alleviating symptoms compared to current therapies, since the phenotype of psoriasis and atopic dermatitis can be heterogeneous due to differences in both gene and environment interactions.

In conclusion, nanocarriers enhance the delivery of a number of drugs into skin. They are a perfect candidate to enhance the treatment of skin disease due to the barrier disruption and close proximity of the target cells. Beyond enhancing the efficacy of current therapies, nanocarriers provide transport for larger drugs and even siRNA, which normally do not penetrate skin. Lastly, while the focus of this review was nanocarriers and skin disease, TDD may aid other conditions via systemic drug delivery through skin. 

## Figures and Tables

**Table 1:** Summary of all in vitro and in vivo efficacy studies for AD or psoriasis nanocarrier systems. DNFB: dinitrofluorobenzene; SDS: sodium dodecyl sulfate.

Reference	Model System	Nanocarrier Formulation	Findings
[97,98]	DNFB-induced AD mouse model	Tacrolimus loaded 20–100-nm lipid nanocarriers	Increased skin targeting of drug and decreased AD like symptoms in mouse model
[99]	Imiquimod-induced psoriasis mouse model	Tacrolimus loaded 150–200-nm liquid crystalline nanoparticles	Increased skin penetration and effectiveness treating psoriasis model compared to drug without nanocarrier
[102]	Nickel sulfate-induced dermatitis model in Wistar rats	Clobetasol propionate loaded 200-nm lipid core nanocapsules	Better controlled drug release and improved skin outcomes compared to drug without nanocarrier
[103]	DNFB-induced AD model in Wistar rats	Betamethasone valerate and diflucortolone valerate loaded 220–350-nm liposomes	Better dermal outcomes with a 10 times reduced dose compared to drug without nanocarrier
[109]	Mouse tail model of psoriasis	Tretinoin loaded <200-nm nanostructured lipid carriers and liposomes	Increased orthokeratosis observed in the mouse tail, indicative of better psoriasis outcomes
[114]	Imiquimod-induced psoriasis mouse model	Methotrexate loaded 278-nm nanostructured lipid carriers	Decreased psoriatic area and severity index
[116]	Ovalbumin-induced AD mouse model	Cyclosporin A loaded 73-nm solid lipid nanocarriers	Decreased local IL-4 and IL-5 cytokine levels
[117]	Imiquimod-induced psoriasis mouse model	Ketoprofen loaded 200-nm chitosan nanoparticles	Increased skin penetration and better dermal outcomes compared to drug without nanocarrier
[118]	SDS-induced AD model in SD rats	Ceramide loaded 200-nm chitosan nanoparticles	Ceramide loaded particles displayed efficacy in regenerating the stratum corneum
[119]	DNFB-induced AD model in mice	Silver ion loaded 200-nm nanolipid complex	Silver ions displayed anti-microbial and anti-inflammatory effects in model of AD
[121]	Ovalbumin-induced AD mouse model	Aqueous dispersion of nC_60_	Nanoparticle treatment reduced IgE and cytokine production and led to better histological skin outcomes
[122]	Ovalbumin and Staphylococcal enterotoxin B-induced AD mouse model	Nanosized zinc oxide particles (<50 nm)	Decreased ear swelling responses, but heightened systemic IgE levels
[123]	Human keratinocytes and hairless mouse skin (SKH1-E)	EGFR siRNA surrounding a 13-nm gold nanoparticle	siRNA linked nanoparticles led to reduction of EGFR both in culture and after topical delivery in the mouse
[124]	Psoriatic and healthy human skin biopsies	Beta-defensin 2 siRNA loaded 100-nm liposomal nanocarrier	Proof of concept beta-defensin 2 knockdown in in vitro keratinocytes from psoriasis patients
[127]	TritonX-100-induced dermatitis in reconstructed human epidermis and porcine ears for penetration analysis	IL-6 siRNA loaded 200-270-nm liquid crystalline nanodispersions	Observed penetration of IL-6 siRNA nanocarriers and the subsequent decrease in IL-6 extracellular release
[128]	DNFB-induced AD mouse model	RelA siRNA loaded nanosized protein carrier	Decreased ear swelling responses, indicating better dermal outcomes
